# Hair coat properties of donkeys, mules and horses in a temperate climate

**DOI:** 10.1111/evj.12775

**Published:** 2017-11-08

**Authors:** B. Osthaus, L. Proops, S. Long, N. Bell, K. Hayday, F. Burden

**Affiliations:** ^1^ Department of Psychology, Politics and Sociology Canterbury Christ Church University Canterbury Kent UK; ^2^ Centre for Comparative and Evolutionary Psychology Department of Psychology University of Portsmouth Portsmouth UK; ^3^ Mammal Vocal Communication and Cognition Research Group School of Psychology, University of Sussex Brighton UK; ^4^ The Donkey Sanctuary Sidmouth Devon UK

**Keywords:** equine welfare, coat properties, thermal insulation, environmental adaptation, fur

## Abstract

**Background:**

There are clear differences between donkeys and horses in their evolutionary history, physiology, behaviour and husbandry needs. Donkeys are often kept in climates that they are not adapted to and as such may suffer impaired welfare unless protection from the elements is provided.

**Objectives:**

To compare some of the hair coat properties of donkeys, mules and horses living outside, throughout the year, in the temperate climate of the UK.

**Study design:**

Longitudinal study.

**Methods:**

Hair samples were taken from 42 animals: 18 donkeys (4 females, 14 males), 16 horses (6 females, 10 males) and eight mules (5 females, 3 males), in March, June, September and December. The weight, length and width of hair were measured, across the four seasons, as indicators of the hair coat insulation properties.

**Results:**

Donkeys’ hair coats do not significantly differ across the seasons. All three measurements of the insulation properties of the hair samples indicate that donkeys do not grow a winter coat and that their hair coat was significantly lighter, shorter and thinner than that of horses and mules in winter. In contrast, the hair coats of horses changed significantly between seasons, growing thicker in winter.

**Main limitations:**

The measurements cover only a limited range of features that contribute to the thermoregulation of an animal. Further research is needed to assess shelter preferences by behavioural measures, and absolute heat loss via thermoimaging.

**Conclusions:**

Donkeys, and to a lesser extent mules, appear not to be as adapted to colder, wet climates as horses, and may therefore require additional protection from the elements, such as access to a wind and waterproof shelter, in order for their welfare needs to be met.

## Introduction

It is estimated that there are currently about 8900 donkeys (*Equus asinus*) 1, an unknown number of mules (*E*. *asinus* ×* caballus*) and just under 1 million horses (*Equus caballus*) 2 in the UK. DNA research indicates that the *E. asinus* line separated from the *caballus* line around 3.4–3.9 million years ago 3. The domestic donkey originates from two different African ass subspecies (*Equus africanus africanus* and *Equus africanus somaliensis* 4). Thus, donkeys’ natural ranges did not reach as far north as those of prehistoric horses 5. The earliest finds of domesticated donkey bones in Europa date from around 800 BC, whereas horses were domesticated around 4000 BC 6. It can be assumed that contemporary donkeys have evolved for warmer and far drier climates than northern Europe and have changed little during the process of domestication. Donkeys are more likely to suffer from hypothermia than horses in the same weather, husbandry and health conditions 7. The skin of a donkey also has different properties to that of a horse, including higher susceptibility to certain dermatological diseases 8. Yet, there are no studies on the properties of donkey and mule hair that would provide an objective background to judging their shelter and welfare needs.

The property of a mammal's hair coat can significantly influence thermal insulation 9 and has therefore a direct effect on their health and welfare. The insulation properties of a hair coat are affected by the thickness of the hair layer, the hair weight and diameter 10, and by external factors such as air movement 11 and moisture 12. The existing literature on the properties of horse hair is very limited. It has been described as 60–100 μm thick and 2–3 cm long (N = 8) 11. A paper on horses housed outdoors under Nordic winter conditions lists the average neck coat length of 10 horses as 4.6 ± 0.9 cm 13. A detailed study of the hair weight of three types of horses (light, warmblood and coldblood) and ponies across the four seasons found that all four types of *E. caballus* grew a winter coat, indicated by a significant increase in hair weight between August and March. The weight of the ponies’ hair increased by over 200% from August to October 14. Our study aimed to provide the first, comparative scientific data on the hair coat properties of donkeys, mules and horses in a temperate climate.

## Materials and methods

### Subjects

Hair samples were taken from 42 animals: 18 donkeys (4 females, 14 males), 16 UK‐native cold blood horses/ponies (subsequently referred to as horses throughout the paper) (6 females, 10 males) and eight mules (5 females, 3 males). The winter clipping data from one mule are missing due to a technical error. The ages ranged from 5 to 31 years, with a mean of 14.5 years (standard deviation [s.d.] 6.7). The mean age for donkeys was 11.9 years (s.d. 6.8), for horses 17 years (s.d. 6) and for mules 15.3 (s.d. 6.1). These differences were not significant (P = 0.08). Only animals that were comfortable with being handled and clipped were included. All animals were socially housed and had 24‐h access to both the outside and a large barn or shelter. No animals were clipped or rugged during the winter and subjects had no known health problems, including PPID.

### Methods

Samples for this cross‐sectional survey were taken in June, September, December 2015 and March 2016. The hair clippings and samples were taken from the midneck about 5 cm below the base of the mane. A 5 × 5 cm area of hair was removed using Liveryman equine clippers and the clippings from each animal were collected and sealed individually in plastic Petri dishes, oven‐dried at 40°C for 12 h in metal wells, and then weighed on scales accurate to 1 mg. The overall weight was calculated as mg/cm^2^. The length and the width of individual hairs were determined from a random sample of five pulled strands, including the roots taken from the neck adjacent to the clipped area. The width of each hair was assessed using a Motic 1820 LED cordless compound microscope, using a 0.1 mm stage micrometer to calibrate the eyepiece reticule for accurate measurement. Results are reported in micrometres (μm). The clippings and strands of the following season were taken from a previously untouched area on the neck.

### Data analysis

For each subject, the hair was weighed, and the mean average from five samples was calculated per season for hair length and width. Data were reported as mean ± s.d. with 95% confidence intervals (CI). We used SPSS 23 for our statistical analyses. Mixed ANOVAS were used to assess differences between the equid types and the effect of seasons. Pairwise comparisons between the equid types were conducted using Tukey HSD corrections for unequal group sizes. We used the Greenhouse–Geisser correction when the assumption of sphericity was not met. To assess any potential sex differences, a series of unrelated *t* tests were performed. The relationship between age and hair coat properties was assessed using a series of bivariate correlations. All tests were two‐tailed.

## Results

### Hair weight

**Figure 1:**
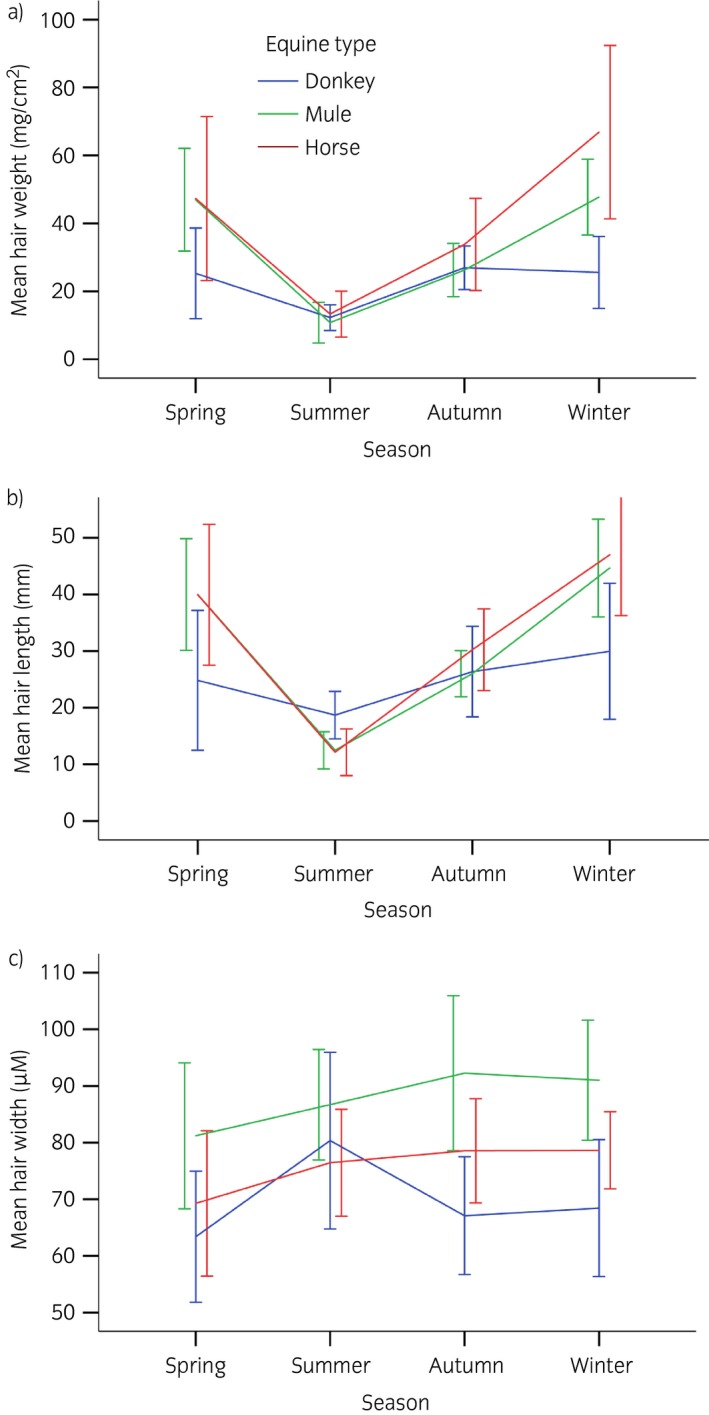
Mean (±s.d.) hair weight, length and width across the seasons for each equid type.

**Figure 2:**
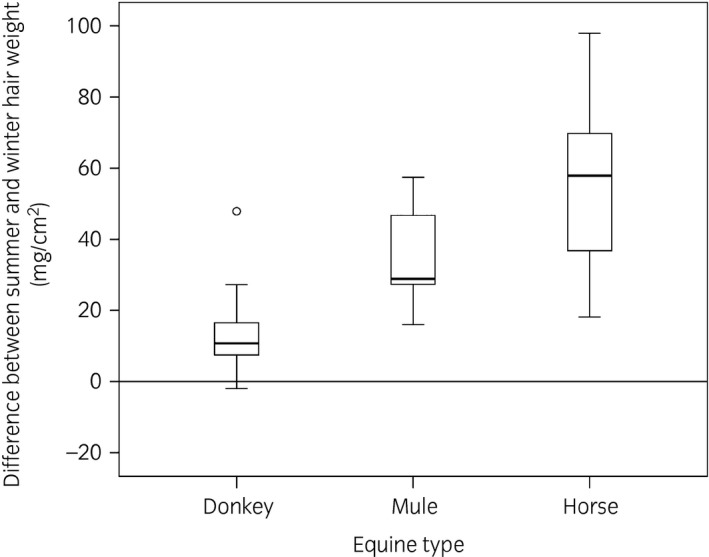
Mean difference (±1s.d.) between summer and winter hair weight for each equid type.

The hair weight across all equid types and season ranged from 2.68 mg/cm^2^ (mule/summer) to 108.72 mg/cm^2^ (horse/winter) (Table 1). There was a significant overall difference between the equid types in average hair weight (P<0.001). A pairwise comparison revealed a significant difference between donkeys (22.52 ± 10.90; 95% CI 19.29–25.75) and horses (40.313 ± 27.117; 95% CI 31.81–48.82) (P<0.001). The difference between mules (34.687 ± 5.689; 95% CI 29.43–39.95) and horses was not significant (P = 0.8), and neither was that between donkeys and mules (P = 0.06). The seasons had a significant effect on hair weight (P<0.001), and the equine types were affected differently (P<0.001). Table 2 shows the pairwise comparison between types, for each season (after Tukey's HSD corrections). There were significant differences in spring and winter between donkeys and horses, and between donkeys and mules. Horses and mules did not differ significantly in any season (Fig 1a). Subtracting the individual summer hair weight from the winter hair weight revealed a significant difference in the weight change between the three types (P<0.001). The donkeys’ hair weight (except for one individual) only slightly increased from summer to winter. There was a small increase for mules and a larger one for horses (Fig 2). Significant differences existed between donkeys (13.30 ± 11.10; 95% CI 7.78–18.82) and horses (53.00 ± 22.93; 95% CI 41.31–65.74) (P<0.001), and donkeys and mules (35.77 ± 14.71; 95% CI 22.17–49.37) (P = 0.02), but not between mules and horses (P = 0.09).

**Table 1:** Descriptive statistics for hair weight, length and width per equid type and per season

	Spring		Summer		Autumn		Winter	
	Mean	s.d.	Mean	s.d.	Mean	s.d.	Mean	s.d.
Weight (mg/cm^2^)								
Donkey	25.30	13.35	22.26	3.77	26.94	6.40	25.56	10.59
Mule	46.95	15.11	10.80	6.00	26.27	7.84	47.73	11.15
Horse	47.31	24.14	13.31	6.74	33.80	13.58	66.84	25.50
Length (mm)								
Donkey	24.83	12.35	18.71	4.20	26.38	8.00	29.98	12.02
Mule	40.00	9.87	12.50	3.28	26.03	4.08	44.68	8.62
Horse	39.96	12.43	12.15	4.12	30.25	7.21	47.00	10.72
Width (μm)								
Donkey	63.36	11.60	80.35	15.60	67.08	10.40	68.42	12.09
Mule	81.19	12.89	86.69	9.75	92.25	13.68	91.00	10.61
Horse	69.25	12.84	76.44	9.45	78.53	9.20	78.63	6.81

**Table 2:** Pairwise comparisons for each season

	Spring		Summer		Autumn		Winter	
	Mule	Horse	Mule	Horse	Mule	Horse	Mule	Horse
Weight								
Donkey	**0.02**	**0.004**	0.8	0.8	>0.9	0.1	**0.02**	**<0.001**
Mule		>0.9		0.5		0.2		0.06
Length								
Donkey	**0.01**	**0.002**	**0.002**	**<0.001**	>0.9	0.3	**0.009**	**<0.001**
Mule		>0.9		>0.9		0.4		0.9
Width								
Donkey	**0.004**	0.4	0.5	0.6	**<0.001**	**0.01**	**<0.001**	**0.02**
Mule		0.08		0.2		**0.01**		**0.02**

P‐values in bold represent a significant difference.

### Hair length

The hair length (in mm) ranged from 6.6 (horse/summer) to 72.2 (horse/spring) and differed significantly between equid types (P = 0.006) (Table 1). Post hoc analyses revealed a significant difference between donkeys (24.98 ± 10.37; 95% CI 21.47–28.48) and horses (32.34 ± 15.95; 95% CI 28.84–35.84) (P = 0.002), but not between mules (30.80 ± 4.41; 95% CI 27.12 and 34.48) and donkeys (P = 0.1), nor mules and horses (P>0.9). The same seasonal pattern was found for hair length as for hair weight. Both mules and horses showed large seasonal changes, with an increase in winter (Fig 1b). For donkeys, the change over the year was less pronounced than for the other two equids. There was a significant overall effect for season (P<0.001), and a significant interaction between seasons and equid types (P<0.001). The donkeys’ hair length showed almost no change across the seasons. In spring and winter, horses and mules had significantly longer hair than donkeys, whereas in summer the opposite was found. All other comparisons showed no significant differences (Table 2).

### Hair width

The hair width (in μm) ranged from 40.5 (donkey/spring) to 113 (donkey/summer). There was a significant difference between the types (P<0.001) (Table 1). Mule hair width (87.78 ± 7.13; 95% CI 81.82–93.74) was significantly different to donkey (69.8 ± 8.04; 95% CI 65.81–73.80) (P<0.001) and to horse hair (75.71 ± 6.65; 95% CI 72.17–79.25) (P = 0.002) but donkey and horse hair did not differ in width (P = 0.08). Season had a significant effect on the width of the hair (P<0.001). The change in seasons affected the equids differently (P = 0.01) (Fig 1c). Throughout the seasons mules had the thickest hair, but in summer there were no significant differences between the types (Table 2). In spring, there was a significant difference between mules and donkeys, but none between mules and horses nor between donkeys and horses. In autumn and winter, all types differed significantly.

There were no differences in hair weight (P = 0.6), length (P = 0.1) and width (P = 0.9) of female compared with male animals. Age was not correlated with any of the hair property measurements (age/weight: r = 0.30, P = 0.06; age/length: r = 0.29, P = 0.07; age/width: r = 0.24, P = 0.1).

## Discussion

Our results clearly demonstrate that there are significant differences in the hair coat properties of these donkeys, mules and horses living in the UK across all measures. Overall, donkeys had significantly lower hair weight and hair length than horses, both in winter and in spring. There were large seasonal changes in hair weight and length for both horses and mules, but not for donkeys. The data for the width differed as mule hair was the thickest throughout the year, with horses taking the middle‐ground, and donkeys with the thinnest hair apart from the summer measurement. Thus, all three measurements of the insulation properties of the hair samples (weight, length and width 9) indicate that donkeys do not grow a winter coat. This is the first study of the hair coat properties of donkeys in any environment and indicates that donkeys are less well adapted to the UK winter than horses and mules.

This is also the first exploration of the hair properties of mules. The hair coat properties of mules were much closer to those of horses than of donkeys. Studies have shown mules to have hybrid vigour in some traits, be intermediate in others, and be inferior to both parent species in others 15, 16, 17. In general, the results here suggest that their hair coat properties are intermediate between their parental species. The fact that mules had the hair with the widest diameter is curious but this result should be viewed with caution due to the rather small sample size (N = 8).

The samples taken from the UK horses align with previous findings. Nordic horses had an average hair length of 4.6 ± 0.9 cm, the mean in our sample was 3.23 ± 1.6 cm 13, while 11 measured 2–3 cm. The same author found hair widths between 60 and 100 μm, we found 75.71 (s.d. = 6.65). Our sample can therefore be seen as representative of the horse population in a temperate climate and provides the first results relevant for this large equine population in the UK specifically. Our data clearly suggest that the native horses studied are better adapted to the temperate climate of the UK, where there are distinct seasonal changes in climate and cool winters. The growth of a thick winter coat provides important protection from the elements. It would be of interest to explore the seasonal hair coat properties of these equid types in tropical climates to assess the extent to which the horses are plastic in hair coat growth and how they compare to donkeys in hotter environments. It would also be useful to explore further hair properties that contribute to thermal insulation, such as oil content, hair shaft structure and the distribution of different hair types, such as wool, guard and tactile hair, across all equid types.

In conclusion, the common perception of donkeys is of a hardy equid, capable of enduring challenging environments. Donkeys are indeed highly adapted to the harsh, semiarid environments that their ancestors inhabited. It would, however, be wrong to assume that this hardiness allows them to thrive under all conditions. What is clear from the results of this study is that donkeys are not able to substantially adjust their hair coat weight, hair length and width in response to colder, winter weather. Thus, these data support the need to provide separate welfare guidelines for donkeys and horses residing in temperate climates, outlining the shelter and management of the two different equid types separately, and affording greater protection to donkeys, potentially by ensuring the availability of wind and waterproof shelters. The intermediate nature of mule hair coat properties should also be considered.

## Authors’ declaration of interests

No competing interests have been declared.

## Ethical animal research

The Donkey Sanctuary's and Canterbury Christ Church University's ethics committees approved the protocol and their representatives gave informed consent for their animals’ inclusion.

## Source of funding

The study was funded by a Donkey Sanctuary Research Grant awarded to Britta Osthaus and Leanne Proops.

## Authorship

B. Osthaus, L. Proops and F. Burden contributed to the study design, study execution, data analysis and interpretation, and preparation of the manuscript. S. Long, N. Bell and K. Hayday contributed to the study execution. All authors gave final approval of the manuscript.
